# Implementation of a Banana Flour Porridge Supplementation Initiative in a Mozambican Primary School: A pilot descriptive study

**DOI:** 10.4102/jphia.v17i1.1749

**Published:** 2026-06-08

**Authors:** José Braz Chidassicua, António Titosse, Amílcar Magaço, Reginalda Mondlane, Edson Mambuque, Maria Patrícia Gonçalves, Réka Maulide Cane

**Affiliations:** 1 Collaborative Researchers’ Team, Sérgio Gago Foundation, Maputo, Mozambique; 2 Ministry of Health, National Institute of Health, Maputo, Mozambique; 3 Ministry of Health, Maputo, Mozambique; 4 Manhiça Health Research Center (CISM), Manhiça, Mozambique

**Keywords:** school feeding, schoolchildren, child nutrition, pilot study, food security, Mozambique, sub-Saharan Africa

## Abstract

**Background:**

Child malnutrition remains a significant public health challenge in Mozambique, particularly in food-insecure rural communities. Locally available and culturally accepted foods may contribute to strengthening school feeding strategies.

**Aim:**

This pilot study described the nutritional status of schoolchildren in a Banana Flour Porridge Supplementation Initiative and assessed the feasibility of implementing and monitoring it in a public-school setting.

**Setting:**

This study was conducted in a rural primary school in Ka Tembe District, Maputo Province, Mozambique.

**Methods:**

A descriptive pilot study was conducted among 65 children aged 6–14 years. All participants received banana flour porridge daily for 30 days. Anthropometric measurements (weight, height, body mass index [BMI]) were collected at baseline, day 15 and day 30. A structured questionnaire assessed dietary habits. Data were analysed using descriptive statistics.

**Results:**

At baseline, most children had adequate nutritional status, with low consumption of dairy and fortified foods, suggesting potential micronutrient gaps. During the 30-day supplementation period, the proportion of children classified as having an adequate BMI increased from 62.5% to 84.4% (aged 6–9 years) and from 57.6% to 87.9% (aged 10–14 years). These are descriptive observations. No adverse effects were reported. The initiative was easily integrated into the school routine and well accepted by students.

**Conclusion:**

Banana flour porridge supplementation is operationally feasible and acceptable in a school setting. Findings should be interpreted as preliminary and hypothesis-generating.

**Contribution:**

This pilot provides early insights into the feasibility and acceptability of incorporating banana flour porridge into school feeding programmes.

## Introduction

According to the 2023 Global Hunger Index (GHI) report, Mozambique was among seven countries that achieved reductions in hunger scores between 2015 and 2023.^1^ Despite this progress, Mozambique continues to face challenges related to high levels of hunger and undernutrition. In 2015, the country ranked 113th globally on the GHI with a score of 37.^1^ While global progress in reducing hunger has been observed, Mozambique still faces persistent challenges, particularly among young children and school-aged populations.^1,2,3,4^

Malnutrition, which includes both undernutrition and overnutrition, refers to a deficiency, excess or impaired utilisation of one or more essential nutrients.^5^ In schoolchildren, undernutrition manifesting as wasting, stunting, underweight and micronutrient deficiencies is influenced by socio-economic, environmental and health-related determinants.^5,6^ Malnutrition among schoolchildren presents significant challenges to their well-being and educational outcomes in developing countries.^6^

According to the Global Nutrition Report, malnourished children are less likely to perform well in school and are more likely to contract infections and chronic illnesses in adulthood.^7^ School feeding programmes support students’ intellectual development.^8^ Malnutrition is a major determinant of impaired mental development and poor academic performance, increasing risks of absenteeism, early dropouts, low enrolment and unsatisfactory classroom outcomes.^5,9^ The Second Sustainable Development Goal aims to end all forms of malnutrition by 2030, including reaching the internationally agreed targets on stunting and wasting in children under 5 years of age.^10^ The coronavirus disease 2019 (COVID-19) pandemic may have worsened this scenario and may compromise the achievement of these goals.^11^

In Mozambique, child malnutrition represents a high economic burden, with an estimated annual cost of approximately 62 billion Meticais (approximately $1 billion United States dollars [USD]), equivalent to 11% of the gross domestic product (GDP).^12^ In 2011, about 43% of Mozambican children suffered from chronic malnutrition, and 8% experienced acute malnutrition.^13^ In 2015, more than three million children under 59 months attending health facilities nationwide presented clinical conditions associated with malnutrition, generating an estimated cost of approximately $118 million.^12^ Chronic malnutrition is associated with delays in school education, with affected children falling behind an average of 4.7 years, and contributing to 19% of class repetitions.^12^ Given this high burden of child malnutrition, interventions targeting school-aged children are urgently needed.^5,14^

School-based nutritional interventions are among the most evidence-supported strategies for addressing child malnutrition in low- and middle-income countries. A 2024 Cochrane systematic review by Spiga et al.^15^ evaluated interventions to prevent obesity in children aged 5 years to 11 years. The review found that multicomponent school-based programmes combining dietary modification, physical activity and nutrition education produced the most consistent reductions in body mass index (BMI) and improvements in dietary quality, while single-component, short-term interventions had limited and inconsistent effects. These findings reinforce the importance of contextualising any anthropometric changes observed in single-arm pilot studies such as the present one within a broader evidence base and support the call for future controlled, multicomponent evaluations.

Banana is one of the most consumed fruits in the world, being produced in most tropical countries and representing the fourth source of energy after corn, rice and wheat.^16,17^ On the other hand, banana flour is a source of energy and rich in nutrients, being especially suitable for children, young people, growing adolescents and people who need to regain weight.^16,17^

Green banana flour (GBF) is produced by drying and milling unripe bananas and is rich in complex carbohydrates, dietary fibre and resistant starch and phenolic compounds.^18,19^ Resistant starch content ranges between 17% and 30% depending on the ripeness stage and processing method, and this fraction is largely preserved in dried flour produced from the whole fruit, including both pulp and peel.^19^ A recent study by Viana et al.^19^ demonstrated that mixed pulp and peel GBF contains significantly higher concentrations of total dietary fibre, phenolic compounds and antioxidant activity than pulp-only flour, with favourable technological properties for food applications, supporting its use in fortified staple foods in resource-limited settings. Depending on the processing method, the flour may thus be produced from the pulp alone or from the whole fruit, with the whole fruit product offering a superior nutritional profile. Because bananas are widely cultivated in Mozambique and relatively inexpensive, GBF could serve as an accessible ingredient for school feeding programmes or nutritional supplementation in low-resource settings.

The consumption of banana flour also contributes to the fight against malnutrition, as well as to the prevention of diarrheal diseases, chronic and cardiovascular diseases and osteoarticular diseases.^17,18,20,21^

Despite these potential benefits, to our knowledge, banana flour supplementation has not previously been tested among schoolchildren in Mozambique, and no local data exist on its impact on the nutritional status of children. The lack of evidence represents a critical gap in understanding how banana flour consumption relates to the nutritional profile of school-aged children in this context. The presence of ‘hunger spots’ in the municipal district of Katembe (Maputo Province, Mozambique), the wide availability of bananas at a relatively low cost and their incorporation into local dietary habits motivated the Sérgio Gago Foundation to implement the ‘Banana Flour Porridge Supplementation Initiative’. Porridge was selected as a suitable vehicle for supplementation because of its ease of preparation, acceptability among children and compatibility with school meal routines. In this context, Complete Primary School of Mutsekwá was appointed by the Ministry of Education and Human Development (MINEDH) to carry out our study and to distribute, free of charge, a package of instant banana-based flour for 1 month. Students consumed the flour as porridge during the school break time to complement their school lunch.

This pilot study aimed primarily to evaluate the feasibility of implementing and monitoring a banana flour porridge supplementation initiative in a public-school setting. Furthermore, it also descriptively characterised the nutritional status of participating schoolchildren during the supplementation period.

## Research methods and design

### Study design and setting

From October 2017 to November 2017, we conducted a pilot, descriptive study at the Complete Primary School of Mutsekwá, located in the Ka Tembe Municipal District, Maputo Province, southern Mozambique. This pilot study aimed primarily to evaluate the feasibility of implementing and monitoring a banana flour porridge supplementation initiative in a public-school setting and secondarily to descriptively characterise the nutritional status of participating schoolchildren during the supplementation period. Because this was a single-arm descriptive pilot study without a control group, the design does not allow causal inference regarding the potential effect of banana flour porridge consumption on nutritional outcomes.

Mozambique lies on the southeastern coast of Africa, covering an area of 801 590 km^2^, and is bordered by Tanzania to the north and by Malawi, Zambia, Zimbabwe, South Africa and Eswatini to the west.^22^ The Ka Tembe Municipal District is flanked to the north and east by Maputo Bay, to the south by Matutuíne District and to the west by Boane District.^23^ The Complete Primary School of Mutsekwá was selected by the Ministry of Education and Human Development because of the district’s food-insecurity profile and the school’s student population, which is comparable to that of the planned full-scale study. However, the full-scale study was not subsequently conducted because of a lack of funding.

### Study population and sampling

A census approach was used, including all eligible students aged 6–14 years. A total of 65 students were included in the study, selected from the list of enrolled students in this age group who participated in the Banana Flour Supplementation Initiative of the Sérgio Gago Foundation. Students were eligible for inclusion if they were aged 6–14 years, enrolled in the school, and had parental or guardian consent and child assent; children outside this age range or whose parents or guardians did not provide consent were excluded. The relatively small sample size reflects the total number of eligible students enrolled in the school and limits the external validity and generalisability of the findings.

### Intervention and portion size

The Banana Flour Porridge Supplementation Initiative was implemented by the Sérgio Gago Foundation in collaboration with local education authorities. The initiative aimed to provide a locally produced banana flour supplement to schoolchildren as a complementary snack during school breaks. The banana flour used in this initiative was produced by drying and milling green bananas in Xinavane, Maputo Province, Mozambique, with bananas preferentially sourced from local producer associations.^24^

Each student received one portion of banana flour porridge during the school break period each school day. The porridge was prepared on-site by trained staff by mixing GBF with water and cooking it until a homogeneous consistency was obtained. Each serving contained approximately 250 g of banana flour, corresponding to an estimated 918.4 kcal per portion, based on the nutritional composition of the product (367.36 kcal per 100 g).^24^

The porridge was served in standard soup bowls, which are deep, wide-rimmed bowls designed for soups. These bowls typically have a capacity ranging from 8 oz. to 16 oz., providing adequate space for the porridge and allowing students to consume the full portion comfortably without spilling. Based on the nutritional composition of the flour (Appendix 1), the supplement provided an estimated source of carbohydrates and dietary energy to complement the children’s other usual meals.

### Anthropometric measurements

Anthropometric measurements were performed using a digital scale with an integrated stadiometer (Seca 704, Seca GmbH & Co. KG, Hamburg, Germany), with a maximum capacity of 300 kg. The device includes an automatic BMI calculation function and provides weight measurements with a precision ranging from 50 g to 100 g. The integrated stadiometer has a measurement range from 60 cm to 200 cm, with millimetre (mm) graduation for height assessment. The scale can be powered by both batteries and an electrical power supply, ensuring operational flexibility during field data collection.

### Nutritional status classification

Nutritional status was classified according to BMI-for-age *z*-scores using the reference standards recommended by the Ministry of Health of Mozambique and Food and Nutrition Technical Assistance (FANTA) guidelines. Children were categorised as: Severe malnutrition: BMI for age < −3 *z*-score; normal/adequate: BMI for age ≥ −2 to < +1 *z*-score; overweight/obesity: BMI for age ≥ +1 *z*-score.

### Data collection

Anthropometric measurements (height and weight) were collected at three time points: Baseline (day 0), midline (day 15) and endline (day 30). Body mass index was calculated as weight in kilograms divided by height in metres squared (kg/m^2^). Nutritional status was classified using BMI-for-age criteria according to the reference standards recommended by the Ministry of Health of Mozambique and the FANTA guidelines.^14,23^ A structured nutritional questionnaire was administered to parents or guardians to assess household dietary habits and children’s food-frequency patterns. Socio-demographic data, including child’s age and sex, area of residence and parents’ education and occupation, were also collected. The dietary diversity score (DDS) was calculated as the number of food groups consumed by the child on the previous day.^25^ A DDS ≥ 4 was considered ‘adequate’ and < 4 ‘inadequate’, following approaches used in prior studies.^25^ Data collection was closely monitored to ensure accuracy and completeness. To ensure data quality, all anthropometric measurements were double-checked by trained field researchers, and records were randomly re-verified for entry accuracy prior to analysis.

### Data analysis

Data cleaning was performed to check frequencies, identify missing values and correct any errors. Data were entered and analysed using Statistical Package for Social Sciences (SPSS) version 15.1 (IBM Corp., 2017) and descriptive statistics – including means, standard deviations and percentages – were used to summarise the findings. No inferential statistical tests were conducted because the study was designed as a descriptive pilot investigation rather than an analytical evaluation.

### Ethical considerations

Ethical clearance to conduct this study was obtained from the Ministry of Health National Bioethics Committee for Health (CNBS) (No. 276/CNBS/17). Written informed consent was obtained from parents or legal guardians, and assent was obtained from the children.

## Results

### Socio-demographic characteristics

The socio-demographic characteristics of the study participants are summarised in Table 1. A total of 65 schoolchildren aged 6–14 years were included in this study, with a nearly equal distribution between those aged 6–9 years (49.2%) and 10–14 years (50.8%). Boys represented 56.9% of the total sample. Among the available data, most parents or guardians had primary education (26.2%), followed by secondary education (10.8%). Farming was the most common occupation (27.7%) among parents or guardians. Regarding health conditions, two participants (3.1%) had digestive problems, 2 (3.1%) had respiratory problems and 5 (7.7%) presented other unspecified health issues before the beginning of the study; none presented oedema.

**TABLE 1:** Socio-demographic characteristics of the students.

Characteristics	*n*	%
**Age group (years)**		
6–9	32	49.2
10–14	33	50.8
**Sex**		
Boys	37	56.9
Girls	28	43.1
**Residence area**		
Rural	65	100.0
Urban	0	0.0
**Parent or guardian education level**		
No formal education	4	6.2
Primary education	17	26.2
Secondary or high school	7	10.8
No information	37	56.9
**Parent or guardian occupation**		
Agriculture and livestock farmer	18	27.7
Student	3	4.6
Unemployed	2	3.1
Fisherman	2	3.1
Driver	2	3.1
Bricklayer	2	3.1
Housewife	1	1.5
No information	35	53.8

### Eating practices

Based on parents’ or guardians’ responses, most children (56.9%) were reported to have eaten at least three meals the day before the interview, indicating a regular meal pattern across both age groups. An adequate DDS was observed in 56.3% of children aged 6–9 years, slightly lower than the 57.6% observed in children aged 10–14 years (Table 2).

**TABLE 2:** Food consumed the day before the interview.

Age group (years)	*n*	Fruits (%)	Enriched porridge (%)	Banana flour porridge (%)	Vegetables (%)	Meat and meat products (%)	Fish and fish products (%)	Dairy products (%)	Eggs (%)	Adequate DDS (≥ 4 groups) (%)
6–9	32	68.8	3.1	3.1	100.0	62.5	78.1	9.4	25.0	56.3
10–14	33	54.5	12.1	0.0	100.0	63.6	75.8	21.2	30.3	57.6

Notes: The dietary diversity score indicates how many different food groups were eaten the day before. A score of four or higher was considered *adequate*, and a score below four was considered *inadequate*. Food groups included fruits, vegetables, meat and meat products, fish and fish products, dairy products, eggs, enriched porridge and banana flour porridge.

DDS, dietary diversity score.

**FIGURE 1:**
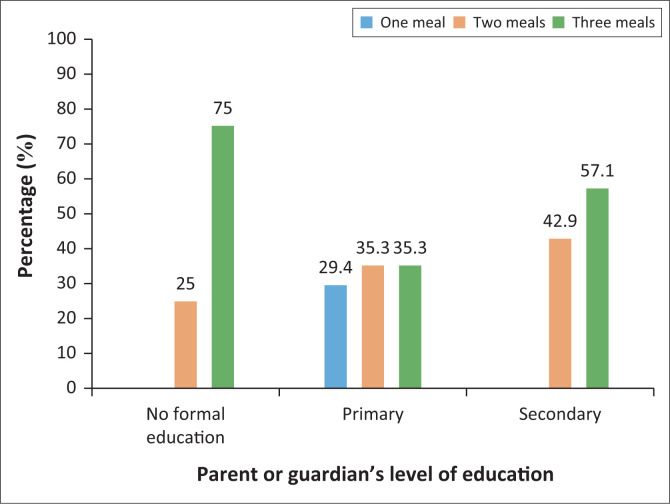
Number of meals given to children by parents’ or guardians’ education level.

**FIGURE 2:**
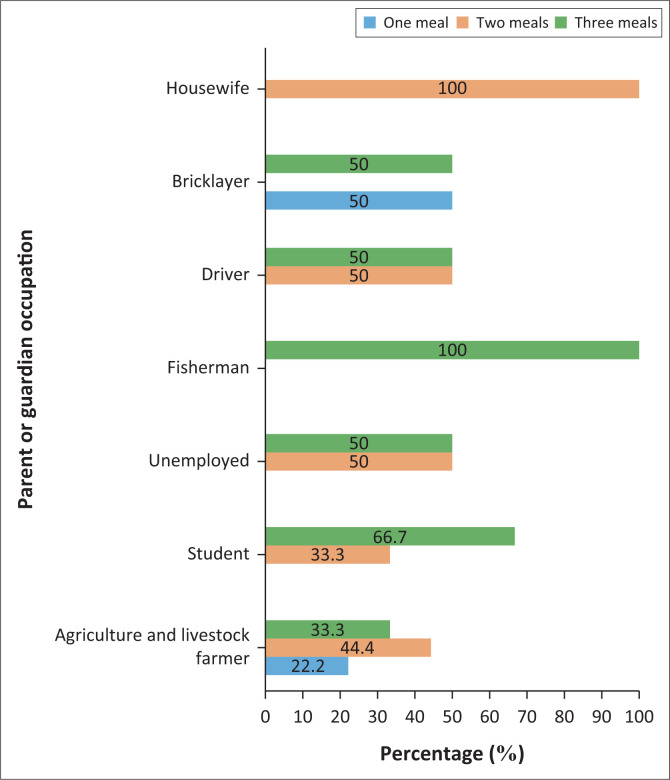
Number of meals given to children by parents’ or guardians’ occupation.

Figure 1 shows that children whose parents or guardians had secondary education were more likely to receive three or more meals per day, while those whose parents had no formal education were more likely to receive fewer meals. Similarly, Figure 2 illustrates that children whose parents or guardians were engaged in agriculture and livestock farming received three or more meals per day compared to those whose parents were unemployed or students.

Table 2 describes the types of foods consumed by the children the day before the interview. All children (100%) consumed vegetables, and more than half consumed fish and fish products (78.1% for children aged 6–9 years and 75.8% for those aged 10–14 years). The consumption of dairy products was low in both age groups (9.4% and 21.2%, respectively). There was also limited consumption of enriched porridge (3.1% and 12.1%, respectively). While 3.1% of children aged 6–9 years consumed banana flour porridge the day before the interview, no consumption was reported among those aged 10–14 years.

Table 3 shows the consumption of liquids among children aged 6–14 years. Nine out of ten children drank water more than once a day (90.6% and 97.0% for children aged 6–9 and 10–14 years, respectively). The percentage of children who consumed juice more than once a day increased with age (15.6% to 27.3%). Overall, there was a high consumption of other types of liquids among both age groups (100% and 93.9%, respectively). Overall, most children achieved an adequate DDS although the low intake of dairy and fortified foods suggests potential micronutrient gaps despite adequate meal frequency.

**TABLE 3:** Liquids consumed the day before the interview.

Age group (years)	*n*	Water > 1×/day (%)	Juice > 1×/day (%)	Milk ≥ 1×/day (%)	Other liquids (%)
6–9	32	90.6	15.6	25.0	100.0
10–14	33	97.0	27.3	27.3	93.9

**Total (rural)**	**65**	**93.8**	**21.5**	**26.2**	**96.9**

### Nutritional status assessment

**FIGURE 3:**
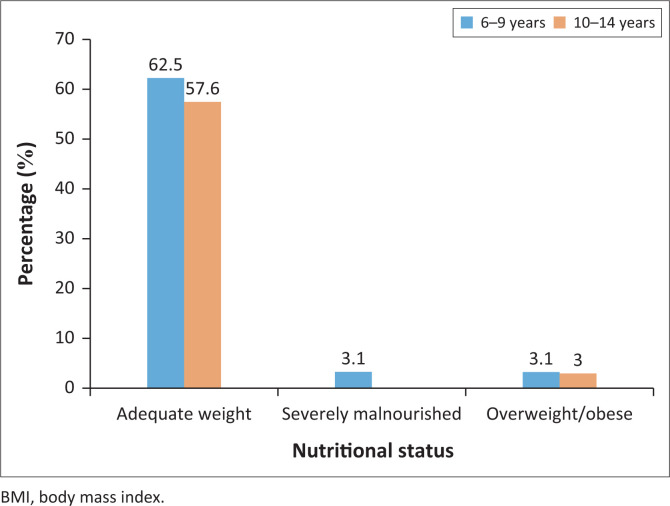
Nutritional status of children before banana flour porridge administration (based on BMI for age z-score). Nutritional status categories: Severe malnutrition = BMI for age < −3 z-score; normal/adequate = BMI for age ≥ −2 to < +1 z-score; overweight/obesity = BMI for age ≥ +1 z-score.

As illustrated in Figure 3, the first measurement showed that most children were within the adequate weight range, with 62.5% of those aged 6–9 years and 57.6% of those aged 10–14 years having normal nutritional status. Among children aged 6–9 years, 3.1% were identified as severely malnourished and 3.1% as overweight (obese), while in the 10–14 age group, 3.0% were overweight and no cases of severe malnutrition were observed.

Table 4 presents the nutritional status of children at each of the three measurement points. In the second anthropometric measurement, an increase of 21.9 percentage points in the proportion of children classified as having adequate nutritional status was observed in the 6–9-year age group and 30.3 percentage points in the 10–14-year age group, compared with the first measurement. The prevalence of severe malnutrition was 3.1% in children aged 6–9 years, with no variation between the first, second and third measurements. There were no cases of severe malnutrition among children aged 10–14 years old. During the 30-day pilot study, all children (100%) consumed the banana flour porridge consistently, and no adverse effects were observed throughout the initiative. The porridge was exclusively prepared and consumed at school during school days and was not distributed to children’s homes; no consumption occurred over weekends. Porridge preparation and consumption were supervised throughout by trained school staff under the oversight of the research team. No banana flour was provided to parents or guardians for home use.

**TABLE 4:** Nutritional status of children before and after consumption of banana flour porridge.

Age (years)	Measurement 1			Measurement 2			Measurement 3			Number of Children (*n*)
	Adequate/normal	Severe malnutrition	Overweight/obesity	Adequate/normal	Severe malnutrition	Overweight/obesity	Adequate/normal	Severe malnutrition	Overweight/obesity	
6–9	62.5	3.1	3.1	84.4	3.1	3.1	84.4	3.1	3.1	32
10–14	57.6	0.0	3.0	87.9	0.0	3.0	87.9	0.0	3.0	33

Note: Percentages are calculated from participants with available anthropometric data at each measurement point. Total enrolled: 32 children aged 6–9 years; 33 children aged 10–14 years. Missing data were mainly because of student absenteeism during measurement sessions.

An increase in the proportion of children classified as having adequate or normal nutritional status was observed between the first and second measurements, and this pattern remained similar in the third measurement. Because of the descriptive design of this pilot study and the absence of a control group, these observations should be interpreted cautiously and cannot be attributed directly to the supplementation. Table 4 presents the nutritional status of children at three different measurements (first, second and third), categorised by age. Overall, the proportion of children classified as having adequate or normal nutritional status increased between the first and second measurements, from 62.5% to 84.4% in the 6–9-year age group and from 57.6% to 87.9% in the 10–14-year age group, and this pattern was maintained in the third measurement. Severe malnutrition remained low (0% – 3.1%) throughout the study, while the prevalence of overweight and obesity stayed stable at approximately 3%. The supplement was consistently consumed by all enrolled children, and the observed increases in the proportion with adequate nutritional status across both age groups should be interpreted as descriptive observations consistent with the feasibility aims of this pilot study and not as evidence of a causal nutritional effect.

Overall, the banana flour porridge was well accepted, and sustained improvements in nutritional status were observed, although causal effects cannot be confirmed because of the study design.

Missing data decreased from 31% to 39% in the first measurement to about 9% in later measurements, reflecting improved follow-up. Missing anthropometric data at baseline were mainly related to student absenteeism during the initial measurement session. The proportion of missing data decreased during follow-up measurements because of improved coordination with school staff and scheduling of data collection sessions.

## Discussion

This pilot study primarily aimed to evaluate the feasibility of implementing and monitoring a banana flour porridge supplementation initiative in a public-school setting and secondarily to descriptively characterise the nutritional status of participating schoolchildren during the supplementation period. To our knowledge, this is the first study in Mozambique reporting the field implementation of a banana flour porridge supplementation initiative among schoolchildren. The initiative demonstrated operational feasibility: All 65 enrolled students consistently consumed banana flour porridge throughout the 30-day period, no adverse effects were reported, and the supplement was successfully integrated into the daily school routine. These findings are relevant in the context of sub-Saharan Africa, where school feeding programmes using locally available foods are recognised as a cost-effective and acceptable strategy to improve nutritional outcomes and school attendance. A recent mixed-methods systematic review by Liguori et al.^26^ across nine sub-Saharan African countries documented evidence of positive nutritional impacts of publicly procured school meals, including reductions in wasting, stunting and underweight, alongside implementation challenges such as insufficient meal diversity, inadequate training and coordination gaps. These findings reinforce the importance of rigorously conducted pilot studies, such as the present one, to validate operational procedures and identify bottlenecks before programme scale-up.^8^ These operational results were further contextualised by the socio-demographic and dietary data collected from caregivers. Parental and guardian education and occupation appeared to influence children’s feeding practices and meal regularity. Children whose parents or guardians had secondary education and were engaged in agriculture or livestock farming were more likely to have three or more meals per day than their peers. These findings are consistent with those reported by Macuácua and collaborators,^27^ which also indicate that parental education and occupation play a crucial role in shaping the dietary patterns of children aged 10–14 years in Mozambique.

Against this socio-demographic backdrop, baseline anthropometric assessment showed that most enrolled children had overall adequate nutritional status, with lower rates of acute undernutrition than typically reported in national surveys of children under 5 years of age.^3,13,28^ This pattern is broadly consistent with the published literature, which indicates that acute malnutrition disproportionately affects younger children, while school-aged children in Mozambique more commonly experience moderate nutritional challenges related to dietary quality rather than total energy deficit alone.^5,6^ The low intake of dairy products (9.4% in the 6–9 year age group and 21.2% in the 10–14 year age group) and fortified foods suggests potential micronutrient gaps that may not be captured by anthropometric measures alone.^29,30^ In Mozambique, micronutrient deficiencies, particularly iron, vitamin A and zinc, remain prevalent among school-aged children and can impair cognitive function, immune response and physical growth independently of energy status.^29^ This finding underscores the need for multi-component nutritional strategies in school settings that address both macronutrient adequacy and micronutrient sufficiency.

Alongside these dietary quality observations, the anthropometric data collected at the three measurement points showed an increase in the proportion of children classified as having adequate or normal nutritional status between the first and second measurements, a pattern maintained at the third. These observations must, however, be interpreted with caution. The single-arm design, without a comparator group, precludes causal inference regarding the anthropometric changes observed. Moreover, a 30-day supplementation period is substantially shorter than the duration typically required for dietary interventions to produce measurable changes in child BMI. Vasques et al.^31^ found that structured diet and physical activity programmes in children and adolescents produce modest and often non-significant effects on BMI, particularly over short follow-up periods, and that meaningful anthropometric change generally requires sustained interventions of several months or more. Changes in BMI for age *z*-score over 30 days may equally reflect natural longitudinal growth trajectories, seasonal variation in dietary intake or measurement variability across data collection rounds rather than any effect of the intervention.^31^ The anthropometric data reported in this study should therefore be understood as descriptive observations during the supplementation period, not as evidence of a nutritional effect attributable to banana flour porridge.

Notwithstanding the absence of causal evidence, the nutritional composition of the banana flour used in this study provides biological plausibility for a contribution to child nutritional health and supports the rationale for future controlled investigation. This flour is derived from whole unripe bananas that are dried and milled prior to peak ripening, a process that preserves a high concentration of resistant starch, a fermentable dietary fibre that escapes digestion in the small intestine and is metabolised by colonic microbiota, conferring prebiotic effects, supporting gut health and contributing to glycaemic regulation.^18^ Munir et al.^18^ demonstrated that GBF contains approximately 30% resistant starch on a dry weight basis and approximately 70% total starch and documented its promising functional properties, including anti-inflammatory, antidiabetic and gut health-promoting effects. Specifically, the fermentation of resistant starch by colonic microbiota produces short-chain fatty acids, particularly butyrate, which support colonocyte integrity, modulate mucosal immunity and contribute to satiety regulation, mechanisms of particular relevance in populations with high burdens of gastrointestinal infection and micronutrient deficiency, such as those in rural Mozambique. Beyond resistant starch, GBF is a meaningful source of potassium, iron, magnesium and dietary fibre (Supplementary File 1), nutrients frequently deficient in the diets of children in low-income settings.^17,21^ These mechanistic pathways, while biologically plausible, remain to be investigated under controlled conditions in this population. Given its low cost, wide availability across Mozambique, cultural familiarity and favourable nutrient profile, GBF is well-positioned as an ingredient for school-based nutritional supplementation programmes. The preparation of the porridge (mixing flour with water and cooking until homogeneous consistency is obtained) is straightforward and replicable in school kitchens without specialised equipment, further supporting scalability.

This potential for scalability is directly relevant to the policy context in which school feeding programmes operate. According to the State of School Feeding Worldwide 2024 report,^32^ the number of children receiving government-led school meals in sub-Saharan Africa rose from 66 million in 2022 to 87 million in 2024, with home-grown school feeding models, which source locally produced foods, recognised as the most sustainable and economically beneficial approach. Banana cultivation is widespread across Mozambique, and GBF requires only low-cost drying and milling infrastructure, suggesting a viable pathway for linking school feeding programmes to local smallholder agricultural value chains. The integration of banana flour porridge into a national school feeding policy framework would address both energy and dietary diversity gaps while simultaneously supporting local food economies. Future studies should therefore incorporate not only nutritional and anthropometric outcomes but also economic analyses of local procurement models, assessments of programme sustainability and measures of dietary diversity at the household level.

School feeding programmes of this kind have been associated with a range of benefits beyond direct nutritional impact, including improved school attendance, reduced cognitive fatigue and greater household acceptance of nutrition-focused interventions.^8^ The strong acceptability observed in this pilot, reflected by complete consumption throughout the study period and the absence of adverse events, is consistent with these expected outcomes. The school-based preparation and delivery model also demonstrated that this supplementation could be operationalised without requiring household compliance, a commonly cited barrier in community nutrition interventions. To our knowledge, no prior study in Mozambique or a neighbouring country has evaluated banana flour porridge supplementation among schoolchildren, which underscores the originality of this work and the need for future controlled research in the region.

### Strengths and limitations

This study has several strengths. Firstly, it represents the first documented pilot field study evaluating banana flour porridge supplementation among schoolchildren in Mozambique, thus contributing novel local evidence in an area where data are scarce.

Secondly, the intervention was implemented in a public-school setting using a culturally accepted, locally available and low-cost food product, which supports the practical feasibility and sustainability of this approach within school feeding programmes.

Thirdly, the study included all eligible students enrolled in the school, reducing the risk of selection bias and providing a comprehensive picture of the nutritional status of this population group.

Fourthly, the study contributed to the validation of data collection tools and field procedures, including anthropometric measurement routines and caregiver dietary questionnaires, which will be directly useful for scaling and designing larger controlled studies.

Fifthly, although the study period was relatively short (30 days), the use of three repeated anthropometric measurements allowed the monitoring of short-term nutritional changes over time. This repeated measures approach improves internal consistency of the observations and demonstrates the feasibility of nutritional monitoring in a school setting, even in the absence of a control group.

An important limitation of this study is the absence of a control group. Although the original main study protocol planned to include one control group, this was not feasible in practice because of the small number of enrolled students (*n* = 65) at the school. Additionally, establishing a control group in a different school would have required resources beyond the scope of the initiative and could have introduced differences in school environment, linked mainly to food security and socio-economic conditions. Furthermore, dividing the children into two groups within the same school was considered ethically inappropriate, as it would have meant depriving part of the students of the supplementary food intervention. For these reasons, the study was conducted as a single-arm pilot descriptive study. These practical and ethical considerations should be considered when interpreting the results, which are not intended to demonstrate causal effects but rather to inform the design of future controlled evaluations.

Additional limitations include potential recall bias associated with caregiver-reported 24-h dietary recalls, the absence of detailed micronutrient intake assessment and the presence of missing anthropometric data at baseline for some participants. These factors should be considered when interpreting the descriptive findings of this pilot study.

## Conclusion

This pilot descriptive study demonstrates the operational feasibility of implementing a banana flour porridge supplementation initiative in a rural Mozambican primary school. Despite an increase in the proportion of children classified as having adequate BMI during the supplementation period, the study design does not allow causal conclusions regarding nutritional effects. Longer-term monitoring and further research are needed to better understand the effects of banana flour porridge consumption on the nutritional status of schoolchildren. Therefore, the findings of this study should be interpreted as preliminary and hypothesis-generating, supporting the need for larger, longer-term controlled intervention studies in this setting. These findings underscore the urgency of designing and funding a full-scale randomised controlled evaluation of banana flour porridge as a school feeding supplement in Mozambique and comparable settings.

## Appendix 1

**TABLE 1-A1:** Information on the nutrition content of the banana flour.

Nutrition content of Processed Green Banana Flour (Finana)	Value
Potassium (mg/100 g)	1224.72
Iron (mg/100 g)	20.04
Protein % (weight/weight N × 6.25)	3.15
Fat (%)	0.88
Ash % (weight/weight)	2.37
Phosphorus (mg/100 g)	0.08
Sodium (mg/100 g)	0.32
Carbohydrates % (weight/weight)	92.49
Energy (kcal/100 g)	367.36
Fiber (g)	8.87
Magnesium (mg/100 g)	30.80
Manganese (mg/100 g)	0.13
